# Qualitative Evaluation of Web-Based Digital Intervention to Prevent and Reduce Excessive Alcohol Use and Harm Among Young People Aged 14-15 Years: A “Think-Aloud” Study

**DOI:** 10.2196/19749

**Published:** 2020-12-15

**Authors:** Laura Elizabeth Tinner, Eileen Kaner, Claire Garnett, Siobhan Mitchell, Matthew Hickman, Rona Campbell, Georgina MacArthur

**Affiliations:** 1 Department of Population Health Sciences Bristol Medical School University of Bristol Bristol United Kingdom; 2 Institute of Health and Society Newcastle University Newcastle United Kingdom; 3 Department of Behavioural Science and Health, UCL London United Kingdom; 4 University of Exeter Medical School Exeter United Kingdom

**Keywords:** public health, adolescent, mobile phone, alcoholic intoxication, internet

## Abstract

**Background:**

In the United Kingdom, despite some downward trends in alcohol use among young people, over one-fifth of young people reported excessive alcohol use in the past month, which is associated with short- and long-term harm to health. Digital interventions to reduce alcohol use, such as websites, among young people present an appealing and cost-effective mode of intervention that can be integrated into the education system. However, relatively few school-based digital alcohol-focused interventions have been developed and evaluated for young people in the United Kingdom.

**Objective:**

This study aims to develop a novel web-based intervention, *Rethink Alcohol*, to prevent and reduce excessive alcohol use and related harm among young people aged between 14 and 15 years, and explore the views of young people, teachers, and youth workers in relation to the content, design, and usability of the intervention.

**Methods:**

Intervention development followed the person-based approach, using theories of social norms and social influence. Qualitative “Think-Aloud” interviews, either one-to-one or paired, were conducted while participants perused and worked through the web-based intervention, talking aloud. Participants included 20 young people (12 female, 8 male), 5 youth workers (4 female, 1 male), 3 teachers (2 male, 1 female), and 1 (male) clinical professional, recruited via youth groups and professional networks. Interviews were audio recorded, transcribed verbatim, and analyzed thematically.

**Results:**

The prototype web-based intervention included normative feedback, information, a quiz, interactive activities, and scenarios. On a rating scale of impressions from poor (1) to excellent (5), participants gave an average score of 3.6/5. A total of 5 themes were identified: content, credibility of the website, making the website easy to understand, design and navigation, and suitability for the audience. These themes reflected views that the content was interesting, credible, informative, and embodied a neutral and nonjudgmental tone, but stronger messaging was needed regarding social pressures and short-term risks regarding safety and risk behavior alongside clarity around pathways of risk; credibility and trustworthiness of information were critical features, determined in part, by the professionalism of design and referencing of sources of information provided; and messages should be succinct and come to life through design and interactive features.

**Conclusions:**

Together, the data illustrated the importance and challenge of communicating nuanced alcohol-focused public health messages to young people in concise, clear, nonjudgmental, and appealing ways. Young people report interest in clear, credible, neutral, and interactive messages regarding social pressures and short-term risks of alcohol use via a web-based intervention. There is scope for optimization and feasibility testing of the *Rethink Alcohol* intervention.

## Introduction

### Background

Although downward trends have been observed in alcohol use among young people aged between 16 and 24 years in the United Kingdom, consumption is higher than for other age groups [1] and over one-fifth report being drunk, in the past month [2]. Digital technologies, such as smartphones, are increasingly integrated into behavior, lifestyle, and education. Approximately 90% of young people in the United Kingdom aged between 16 and 24 years, and 41% of those aged between 5 and 15 years own a smartphone [3,4]. As such, digital interventions could play an important role in changing behavior and reducing alcohol consumption in this group, thus contributing to preventing a range of alcohol-associated short- and long-term harm to health [5-10].

Evidence highlights that universal digital alcohol and other drug prevention interventions can be beneficial in reducing the frequency of alcohol or drug use among both young people and adults [11-18]. One systematic review demonstrated that engagement with personalized digital interventions that aim to reduce hazardous and harmful alcohol use among adults was associated with reduced consumption per week and one less binge drinking session per month, compared with controls [14]. Another review showed that alcohol-focused interventions reduced consumption among college students screening positive for hazardous alcohol use [13]. Across reviews, small but beneficial impacts have been demonstrated within universal and targeted interventions across a range of outcomes including number of drinks per drinking day, number of heavy drinking days, average weekly consumption, and frequency of binge drinking for participants from schools, health care settings, and the community [11,13,14,18,19].

Digital interventions may provide an appealing mode of intervention for young people, while being flexible, adaptable, person-centered, and cost-effective when delivered at scale over time [14,20]. In addition, technology and digital engagement are increasingly integrated into education, with evidence indicating a beneficial impact on educational outcomes if used appropriately to support learning and teaching [21]. The UK government recently published a strategy highlighting the ambition of integrating innovative technology into education to improve efficiency, remove barriers to education, and drive improvement [22].

To date, however, studies have mostly targeted young adults or the general population, and comparatively fewer digital interventions have aimed to reduce alcohol consumption among adolescents [23]. In addition, few universal school- or youth group-based digital interventions have been developed and tested using randomized controlled trials. Globally, 8 have focused on alcohol alone, but none were tested in the United Kingdom [15]. Although a digital intervention for young adults has been developed in England and acceptability was investigated [24], effectiveness has not been established [25,26] and none, to our knowledge, have investigated the development of a digital alcohol-focused intervention designed specifically for use in school and youth group settings.

### Objectives

We aimed to develop a web-based intervention (*Rethink Alcohol*) to reduce excessive alcohol use among young people aged between 14 and 15 years. The intervention aimed to: (1) delay initiation of alcohol use (primary prevention) and (2) reduce the average amount of alcohol consumed per month (secondary prevention). Secondary aims were to reduce risky single occasion drinking, frequency of alcohol-related negative consequences, and changes in perceived injunctive and descriptive peer norms. Here, we report the theoretical basis and stages of intervention development, and the views of young people, teachers, professional experts, and clinicians regarding design, content, acceptability, usability, and implementation of the intervention in practice.

## Methods

### Development of the Digital Intervention

**Figure 1:**
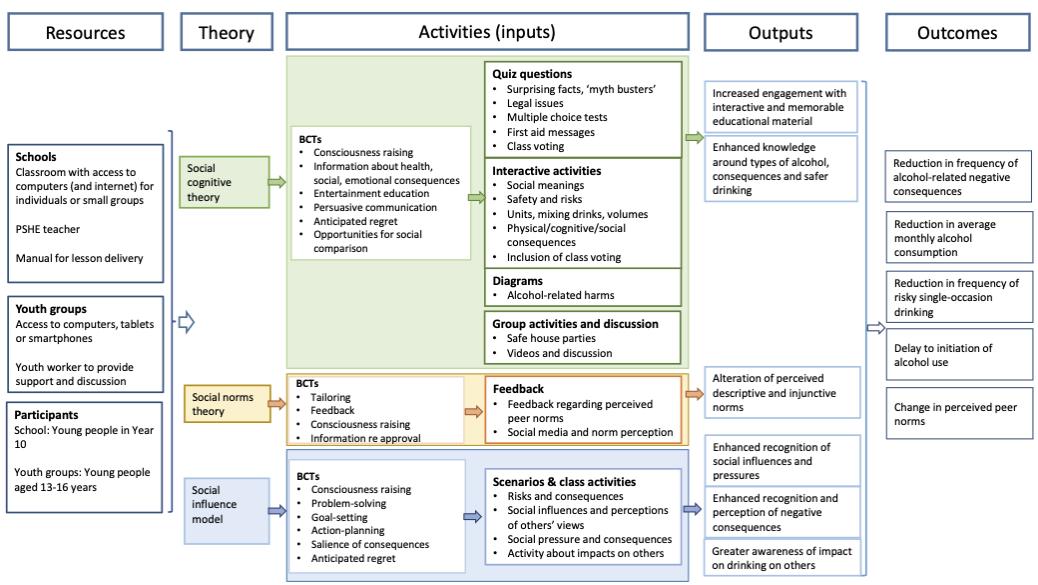
Logic model for intervention development. BCT: behavior change technique; PSHE: Personal, Social, Health, and Economic.

Development of the intervention *Rethink Alcohol* followed the person-based approach [27]. First, a scoping literature review of alcohol-focused digital interventions was conducted. Intervention components, content, duration, theory, and outcomes of alcohol-focused digital interventions for young people were examined, and behavior change techniques (BCTs) shown to be effective were noted [18]. BCTs form part of the *modelling* phase of complex intervention development, which involves hypothesizing *what* to target (behavioral determinants) and *how* to do this (techniques to change these determinants; [28]). Our previous qualitative studies with young people aged between 14 and 15 years, and 18 and 20 years [29,30] highlighted the peer, family, and socio-cultural influences of alcohol use, the behavior change required, and the behavioral and attitudinal challenges to be addressed by the intervention. Figure 1 shows our logic model for the intervention.

Throughout the process of intervention development, we engaged with 3 young people advisory groups (YPAGs): Generation R, ALPHA (Advice Leading to Public Health Advancement), and YPAG north east (YPAGNE). YPAGs provided feedback at each stage, which, alongside qualitative research findings, shaped the written content, activities included, design and layout of the prototype website, and considerations about implementation and use in practice. For instance, in workshops, young people highlighted content that should be included (eg, social pressures, drink driving, managing parties, injury, violence), design features that would be appealing (eg, using a unit calculator, voting on the types of images to include), and the appropriate tone to be used (eg, nonjudgmental, accepting language). Contribution from our steering group, which included 2 young people, also shaped the content. These processes informed the guiding principles of the intervention and its content, theoretical perspective, and design.

*Rethink Alcohol* was based on social cognitive theory, social norms theory, and the social influence model. Intervention components were designed to incorporate these theoretical perspectives and map BCTs to each component. The first version of the prototype website (v1.0) was created using the UXPin software (UXPin Inc). Feedback regarding v1.0 was obtained from YPAGs on topics covered, the written content, name of the website, activities to be included, design, layout and the types of images to use, navigability, tone, and how the website could be used in a classroom setting, enabling the development of a second-stage website (v1.1). Exemplar screenshots of the intervention website are shown in Multimedia Appendix 1.

In terms of content, the prototype website was structured around 4 themes: (1) Reality check: do people my age really drink alcohol? This section included questions regarding the perceived frequency of alcohol intake and getting drunk and feedback based on data from a survey of young people in England. An opportunity for goal setting was included. In addition, a quiz, including questions about alcohol content of different drinks, sugar content, mixing drinks, health consequences, and drink driving. (2) A “Get the facts” page provided information about alcohol and the law, drink driving, action of alcohol in the body, and health consequences. Facts were also included by theme, as advised by young people, so that information could be identified quickly, related to the themes of alcohol and health (addiction, accident and injury, sex and consent, hangovers, cancer, alcohol, and the brain); units, strength, and alcohol by volume; drinking and friendships (including social pressure, first aid); and guidelines for alcohol intake. (3) Top tips for staying safe included content regarding how to look after a friend whose had too much alcohol, the recovery position, guidelines, managing pressures, and sources of information and support (eg, addiction support). (4) Real life, which included scenarios that could be played out by choosing different options for action, such as a house party involving injury and sharing of photos, and outdoor drinking and violence. Our intention was to include videos prepared by young people, but this was not possible within the available timeframe; thus, a format representing messages communicated via social media was used.

### Think-Aloud Study

Qualitative interviews were intended to gain an understanding of views regarding the breadth and relevance of content as well as views regarding navigability and design, hence guiding refinement and thus optimization of the prototype website. The findings will inform a second stage process of altering design and updating content to reflect the views obtained.

### Participants

Young people, teachers, and youth workers were recruited purposively from youth groups and schools in Bristol and the surrounding area, aiming for diversity in socioeconomic status and alcohol use. Snowball sampling was employed to quickly access the target population. Young people were eligible for participation if they were between 14 and 18 years of age at the time of the study. Data from 3 paired interviews could not be included in the analysis owing to an error during fieldwork and individuals outside of the eligible age range (ie, older than 18 years) being included in these interviews. Although the intervention is targeted to young people aged between 14 and 15 years in the school environment, we anticipate that the website could be used in youth groups and other settings, which might include young people with a broader age range. We were also keen to recruit young people from youth groups to include a range of demographic groups from different geographical areas within the timescales of the study. Young people attending youth groups were typically aged between 16 and 18 years. In addition, we considered that it would be helpful to hear from those who are able to reflect on previous experiences and provide helpful feedback about information or support that would have been helpful at that time point in adolescence. The professional participants were approached through existing networks and contacted by email or telephone with an electronic copy of the information leaflet and summary of the study aim.

### Procedures

Semistructured interviews guided by the Think-Aloud method [24,27,31] were conducted by the principal investigator (GM; female), SM (female), or other study staff, all of whom had experience of qualitative interviewing. Interviews were guided by a flexible topic guide and lasted approximately one hour, with the Think-Aloud component included at the start of the interview. The development of the topic guide was informed by related studies [25,31] and our focus was on understanding young people’s views regarding content, design, and usability, as per feedback from YPAGs. Paired interviews (ie, between friends) were conducted if participants preferred. Although the think-aloud section of the interview aimed to identify participants’ views and impressions while they used the website, what they were thinking about and feeling through the process, the semistructured section aimed to provide an opportunity to explore views around aspects of the website, such as perceived accuracy, presentation, appropriateness, and completeness of information provided, if these points had not been covered. The interviewer’s role was to prompt the participant to keep verbalizing their thoughts on the website [24] to explore views on content (eg, the information on the website useful, clear, comprehensive), design (eg, views on the style, font, colors, layout of the website), and usability (eg, is the website easy to navigate). Participants were also asked what they thought of the interactive components included, such as quizzes, scenarios, and personal feedback. Interviews with teachers aimed to explore the applicability of the Personal, Social, Health, and Economic (PSHE) curriculum (eg, educational content, timing) and feasibility of implementation. Participants were made aware of the reason for conducting the study and the research team’s interest in the topic.

Written informed consent was obtained from all participants and parental consent and a young person’s assent obtained for those aged under 16. Adolescent participants were offered a £15 (US $19.88) gift voucher, and teachers and youth workers were offered a £25 (US $33.14) gift voucher to account for their time contribution. The disparity in payment between young people and teachers and professionals reflected the fact that teachers and professionals contributed their time in working hours. Ethical approval was obtained from the University of Bristol Faculty of Health Sciences Ethics Committee (Ref: 74270).

### Analysis

Interviews were audio recorded using an encrypted recorder and transcribed verbatim. We undertook thematic analysis guided by Braun and Clarke [32], adopting a framework approach to organizing the data [33]. The coding framework was developed following the initial reading of a selection of transcripts by 2 researchers (Multimedia Appendix 2). One researcher then applied these codes to the transcripts, refining the codes, and adding new ones as data analysis progressed. Line by line open coding was used to assign qualitative codes to sections of the data that were reflective of that code [34]. The codes were discussed to determine if any extracts needed to be moved, if codes needed more refinement or additional codes should be considered, with disagreements resolved through discussion. Participants did not provide feedback on the findings. The themes presented in the results section represent the cumulation of the coding process. Coding and data management were conducted on NVivo 12.0 (QSR International).

## Results

### Think-Aloud Study

We interviewed 12 participants through single interviews and 17 participants in paired interviews (n=29). The core themes identified in the Think-Aloud study were (1) content, (2) credibility of the website, (3) make the website easy to understand, (4) design and navigation, and (5) suitability for the audience. A total of 26 participants, across all participant groups, gave an overall score out of five for the website ranging from scoring 5 (excellent) and scoring 1 (terrible) with a mean score of 3.63, indicating that they thought the website was good, but some improvements were required. The design of the website was not as anticipated, and many participants appeared to let down by this aspect, which was reflected in their scores. The data obtained from paired interviews did not appear to differ substantially from the data obtained from single interviews. The themes in this section draw upon suggestions for improvement as well as insights about adolescence and alcohol use that were relevant for the development of the intervention prototype.

### Content

In general, participants felt that there was an appropriate amount of content, that the content was accurate and interesting, and that the website was informative. Most of the comments about the content related to how it could be more engaging, if the content should be moved to a different area of the website and ideas for additional content.

Although the content included in the website had been strongly informed by YPAGs, several participants thought there should be more on the website about the consequences of being drunk beyond health implications, such as engagement in risky behaviors (such as taking drugs or swimming in a canal), potential dangers of sexual assault, drink spiking, and getting home safely. This was connected to the idea of preparing young people for what to expect if they choose to get drunk for the first time. Several participants thought there should be signing to support information related to these wider issues in addition to those already included. A common suggestion was to incorporate information about wider risks in the *scenario* section, which many participants engaged with:

Maybe you could have a scenario about someone's drunk, and they're about to have sex or kiss, or something like that. Having a scenario about that, asking, 'Is this appropriate when they have drunk stuff and I have drunk stuff, or they have drunk loads and I have not?'

Participant RO4, young person

Probably, a little bit more around risky behavior, and that just the increases of risky behavior...Your risky behavior, but also the behaviors of other people that can become risky.

Participant R03, youth worker

These comments about additional content are mostly related to being drunk and social situations. Addiction was mentioned by some participants, which while included on the website, indicated that young people think an alcohol intervention should have an awareness of a range of risks. Some participants thought that *scenarios* on the website could form a broader discussion in the classroom around more complex issues such as decision impairment and risky behaviors. These topics are already discussed in PSHE lessons and are perhaps more immediate concerns of young people than the long-term impacts of alcohol on health:

You say, “Well, it’s happened more than you’d think,” and it’s not like you want to show them CCTV, but sometimes there are incidents where they have the movements of that person, and they were on their own. Then that starts another conversation, “Well, why were they on their own? Okay, what happened? Why were they making the wrong decisions, what made them think it would be alright to go in the water?” You know, and then you might get onto, “Oh, well, they were showing off because they were drinking alcohol,”

Participant R09, teacher

In addition to wider risks, most participants saw peer pressure and social influence as important considerations. Some thought that enhancing peer pressure sections on the website would be useful:

It gives you the information, but it does not really say what you can do. It just says ‘your friends will respect you for being true to yourself,’ that’s not always the case. Maybe write an alternative like, ‘if they do not, it’s not healthy. Get out of the group,” sort of thing. If they stay in the group thinking their friends will respect them, that is not always the case.

Participant RO20, young person

I like the little bit of text at the top that sort of summarises the concept of peer pressure… do not want to drink but I feel like I need to fit in with everyone.

Participant RO27, young person

Most participants had positive comments about the quiz section of the website, with almost all participants taking the quiz first. Some suggested that discussing the sugar content of alcohol was a good approach to engaging young people, who may be health conscious but may not have associated alcohol with high levels of sugar. A few participants thought that this section should go further to talk about calories and how alcohol can affect muscle mass.

Overall, participants were generally positive about the content of the website. There were comments about additional areas of importance that could be included, which centered around wider risks and peer pressure. Such issues were thought to be best addressed through interactive elements of the website, such as the scenarios and the quiz.

### Credibility of Website

The credibility, accuracy, and trustworthiness of the website appeared to be crucial for the sample. Most participants thought that the website was a credible and trustworthy source of information based on positive comments about the homepage and fact pages. However, some participants assumed that the information was accurate and from a credible source as it *comes from the university*, indicating that their involvement in the study had resulted in some preconceived ideas. Several participants thought the *look* of the website was critical for credibility, suggesting that a *professional* and *consistent* design would make a website more trustworthy. Simple design elements such as having the same font throughout, the same style of images and using *modern* colors were thought to add a sense of professionalism and therefore enhance trustworthiness and credibility:

I think you have got a lot of relevant stuff here, not all of it relevant, and I think the presentation just could be a bit more professional. I think it’s coming along quite nicely.

Participant RO1, young person

If you clicked on different things, it was not different colours or themes. I thought that it also adds to it. Like trustworthiness, like thought has been put into its design.

Participant RO5, young person

References with links and source information after facts were mentioned by a few participants as things they looked for when establishing the credibility. Some also mentioned the idea of a kite mark or logos of sources from which the information was gathered, as these are things they would generally look out for when viewing a website:

I think there is also a slight bit of distrust if things have not been referenced.

Participants RO11 and RO12, young people

The professional interviewees stated that young people question all the information they receive and are typically critical of where information comes from. Referencing is even more crucial if the facts or statistics are surprising to the young people, such as “23% of 15-year-olds have been drunk in the last month” [35]. One professional participant mentioned that they would expect young people to ask for the source of facts during classroom discussion. Having an easily accessible source reference allows the session to flow better and gives the teacher or youth worker confidence in referencing the facts:

If I was using this resource with young people, it might just be nice to know where that came from, from a professional angle. So young people might come and ask that question and say, “Well, where have they got that from? How do they know that?” So we can just say, “Oh, it was a study by X, Y, Z.”

Participant R03, youth worker

Maintaining these elements thought to enhance credibility could help young people engage with the content easily, as no time is spent questioning the sources and trustworthiness of the website. Overall, trustworthiness, credibility, and accuracy are important to young people and can be achieved through consistent and professional design elements as well as clear transparency around where information comes from.

### Make the Website Easy to Understand

This theme was organized around 3 subthemes: (1) clarity, (2) levels of complexity, and (3) quantity.

Clarity was important to participants, as discussed by both young people and professionals. Clarity might be achieved through wording, phrasing, and consistency of units, such as for alcohol strength:

Obviously, a big bottle of a cider, depending on the size, will have more units of alcohol in than a single shot, so that's a little bit of a confusing message, perhaps.

Participant R03, youth worker

I feel like the question is somewhat unclear. This could mean whether they had too much alcohol this one specific time and they’re drunk, or if they frequently have too much alcohol and they have a problem.

Participant RO15, young person

Using specific brands or sizes of bottles may be one strategy to limit misinformation. Here participants highlighted the potential for confusion by using generic terms such as *alcopops* or *bottle of cider* as there is a wide range of types, sizes, and alcohol strengths. If participants are clear about which drink is being referred to, young people may be able to engage with the content better and learn how drinks compare in terms of alcohol strength, with less time spent questioning what the drink is. One professional mentioned that *alcopops* are not very popular with young people, which was reflected in a few young participants not knowing what *alcopops* are.

There were also examples of complex ways in which alcohol could affect people, with careful thought needed to describe them to avoid giving the wrong information. An example of this is outlining diseases that can result from alcohol consumption. One professional participant addressed how wording could be misleading around the risk of sexually transmitted infections (STIs). They highlighted the need to outline how alcohol-associated behaviors could increase risk rather than the alcohol itself being the direct cause of STIs. Other examples include the need for clarity around the connection between energy drinks and alcohol, and how mixing these drinks may lead to other risky behaviors, as young people may not have considered them to be linked. Finally, more explanation was suggested around how alcohol may increase the likelihood of engaging in a fight, which was a source of confusion for a few participants. One suggestion for adding clarity here was to discuss how alcohol can affect how people make choices, as this is frequently discussed in PSHE lessons.

The quantity of information, namely instances where there was too much written information on a page, was important to both young and professional participants. Although repetition might not impact understanding and some repetition allows information to be received quicker, some participants noted too much repetition would mean that users lose interest. Similarly, *text heavy* pages may discourage young people from reading anything on the page at all. Several participants valued text boxes or bullet points broken up with images:

I think just the smaller quantity of text made it easier to understand for 14- and 15-year-olds.

Participants RO18, young person

This theme of quantity in turn links to getting the information quickly*,* which participants felt was important for some kinds of information more than others. For instance, finding out what to do if a friend is drunk is more time-sensitive than current statistics on age-group drinking.

The most mentioned concept in relation to the present theme was clarity. The quantity of text should be carefully considered, with text broken up by boxes, bullet points, or images. However, this should be balanced with clear descriptions of more complex pathways between alcohol use and outcomes.

### Design and Navigation

Design was important to young people, with many providing positive comments about the design of the intervention, most commonly about the colors, fonts, and the home page. The most frequently mentioned design elements that young people valued were images and videos. Specifically, more *boring* or *serious* elements of the intervention, such as information pages, should have more images or diagrams than other pages. One participant suggested using an image of the body that users could click on to get facts:

Yes, again, more images rather than lots and lots of texts. Break it up a bit more. You know, maybe having pictures of a body and being able to draw lines off it saying stuff that’s affecting this. Being able to click on different parts of the body…It just makes it a little bit more dynamic, I think.

Participant R06, a youth worker

Participants thought it was a combination of aesthetics as well as interactivity that engages young people in delivering health information. Others noted the importance of not having too many different things *going on* within one page. For instance, in contrast to feedback from YPAGs, it was considered that fact boxes should not interfere with their interaction with the quiz or scenarios. Participants also noted the importance of the website being easy to navigate, being able to reach where you wanted to with minimal clicks and with an understanding of where every link would take you. Many of the participants thought the home page was clear and it was easy to see where everything was. Participants also liked having the top ribbon visible on every page.

### Suitability for the Audience

Interactivity was noted by most participants as something that would make the website appropriate for young people. A common suggestion was the use of videos to show the scenarios or to show how to put someone in the recovery position. Participants thought that this would not only be engaging for young people, but it might improve accuracy and clarity of information. Some participants said that some young people have functional literacy issues, so a video would be a better medium for them. Some participants felt that the scenarios section would benefit from being made more engaging, age-appropriate, and fun:

Yes, just a little bit engaging or something, you know? Yes, almost like that little video in the… “They’re on the street outside the club or something, they’ve passed out, so you turn them over like this, and then you do this to get help.”

Participant R05, clinical professional

You know the text message shown? It might be worth doing another option next to it. You know on the first bit where you could click it?

Participant RO19, young person

Wording and tone are crucial for communicating health messages with young people. The youth participants highlighted that they did not want health information to come across as patronizing or too much *from an adult*, although this should be balanced with the trustworthiness and credibility of the information sources. Participants were also mindful of wording that might make them overly worried about alcohol or that tried to imitate the adolescent language. Therefore, information should be delivered with a neutral and nonjudgmental tone. In the main, participants felt that the website embodied this tone. However, some did highlight a few areas that could be perceived as patronizing:

I do think this bit, about how not to get a hangover: ‘It’s easy, do not drink anything’ is a bit condescending. If you’re saying, ‘How to stop a stab wound,’ and it’s like, ‘Easy, just don’t get stabbed,’ you’d be like, “That doesn’t help me.”

Participants RO15, young person

Generally, participants felt that the website was age appropriate; however, including interactive elements would engage young people for longer.

## Discussion

### Principal Findings

Our web-based intervention for young people has the potential to be usable and informative for young people, and to address the determinants of behavior (eg, peer effects) and perceptions around alcohol-associated risk. The key requirements for engagement and usability include professional and consistent design, succinct, text-light messages that clarify pathways of risk, interactive and audio-visual components, and trustworthy information. Our findings are consistent with those of studies exploring the usability of alcohol-focused apps for adults and young adults [31,36], which similarly reported the importance ascribed by users to design, interface, and clarity, with low user input (ie, short blocks of text, videos), and credibility and trustworthiness of the information provided. The importance of trustworthiness is also supported by recent systematic reviews, which have highlighted that a *credible source* was one of the BCTs that predicted alcohol consumption [14]. In addition, although we sought user views before this study and regarding an earlier prototype, we nevertheless identified divergent views around content and design, demonstrating, as for others [36], the challenge of developing a digital intervention that is appealing and/or engaging to all.

In part, this is related to the central consideration apparent throughout the interviews, which is the complexity of alcohol use among young people, the multiplicity of situations where it might occur, and the wide range of types and brands of alcoholic drinks. There is a tension here in that participants acknowledge the complexity around alcohol use, while also wanting limited text on the website in favor of images or videos. Balancing nuance, accuracy, and engagement is thus a second key challenge in communicating about harmful alcohol use with young people.

Using the correct tone seemed to be paramount when communicating about alcohol with young people. Participants were mindful of not wanting to be patronized, judged, scared, or told what to do. Although most participants felt the website embodied the correct tone, this further highlights the difficulty in attempting to reduce harmful alcohol drinking while also providing nonjudgmental information about intoxication and alcohol use. As such, the web-based intervention is balancing the dual aims of maintaining the rejection of harmful alcohol behavior before it starts as well as altering it where it exists [37], which is reflected in the website’s primary and secondary prevention objectives. In the main, participants thought this balance was achieved by preparing young people for potential situations related to alcohol use as well as delivering facts through neutral language. However, any further enhancements to the website should maintain this consideration of tone, which is informative about the negative consequences of harmful alcohol use while also being nonjudgmental.

There is also a wide range of potential consequences or wider risks associated with alcohol use that young people saw as important for a web-based alcohol intervention. This is noteworthy, since systematic reviews have reported no association between the behavior change technique *information on consequences* and reduced drinking frequency [14,18]. This highlights how perspectives on digital intervention are shaped by individual experiences and thus the importance of personalization. It further reiterates the challenge of communicating information to reduce harms alongside that aimed at reducing consumption.

Many young people indicated that alcohol was embedded within more general concerns, such as what they conceived of as peer influence. This is consistent with previous work that found peers play a central role in young people’s drinking behavior and the social contexts within which it occurs [30,38]. The nature of peer influence alters throughout adolescence, with younger adolescents experiencing a greater focus on experimentation, intoxication, and social conformity [29], and may be more susceptible to peer influence [39]. Our findings are consistent with this idea, as participants were interested in sections of the website that addressed peer influence and social situations where they may encounter alcohol use and intoxication. Therefore, health messages related to alcohol use, such as disease and injury, may seem less relevant to younger adolescents, as their more immediate concerns relate to new social situations. Interestingly, young people did not specifically suggest including content regarding the impact of family context, so this was not included. Nevertheless, a parallel qualitative study that explored the views of parents or guardians in relation to adolescent alcohol use (Mitchell S, unpublished data, 2020) will inform the next steps, such as the possibility of a parent-focused section or related resources.

It was apparent that young people had the expectation that websites should be engaging, interactive, and have a contemporary aesthetic. This is unsurprising given how embedded the internet is in people’s everyday lives [40], with young people being high users of new technologies. Increases in smartphone availability and affordability mean instant access to the internet for young people in the West [3]. Horvath et al [41] recommend planning adequate budgets for design elements, and teams should be expanded to include experts in learning technologies or web-based intervention development. As many participants noted the difficulty in keeping young people engaged, attention to interactive media such as videos should be considered for delivering health messages, which in turn may need greater expertise and resources. This corroborates previous work with young people who found that web-based health interventions should be presented in an attractive and interactive way to improve the efficacy of the intervention [42]. We included a range of sections and activities in the web-based intervention so that participants would have the opportunity to engage with different components, incorporating the distinct BCTs outlined in the logic model. In the next phase of this work, we will also develop a clear teaching guide and lesson plan, and guide youth group settings to increase the likelihood that young people will engage with all components.

### Limitations

We had a small sample in 1 geographical area; thus, the findings cannot necessarily be generalized across geographical regions. The sample also covered a broader age range than the intervention was designed so that older participants could reflect on their recent experience. A larger sample of young people aged between 14 and 15 years would have enabled more extensive analysis around the viewpoints of this age group and differences by age and sex, which was not possible in this study. Interestingly, by including this wider age range, we identified that such a resource could be useful for a broader group. Our sample size was pragmatic and limited by the timescales of our study. However, we found that similar themes were raised by young people and consider our sample size sufficient to enable development and optimization of the prototype website [43]. Finally, we did not gather data regarding participants’ alcohol use; thus, we cannot ascertain how alcohol behavior may have shaped perspectives relating to the intervention.

### Conclusions

In this study, we have identified the importance of appealing design, low-input interface, interactivity, and credibility in an alcohol-related web-based intervention and the need for involvement of young people at all stages of design and development to ensure that content, tone, usability, and appeal are optimized. Our study also demonstrated the promise of the web-based intervention *Rethink Alcohol*. The next steps will involve optimization and testing of the feasibility of implementing the intervention in schools and youth group settings.

## Appendix

Multimedia Appendix 1 Screenshots of the intervention website.

Multimedia Appendix 2 Themes and subthemes used to code the interview transcripts during data analysis.

## Abbreviations

ALPHA: Advice Leading to Public Health Advancement
BCT: behavior change techniques
CRUK: Cancer Research UK
NIHR: National Institute for Health Research
PDF: Post-Doctoral Fellowship
PSHE: Personal, Social, Health, and Economic
STI: sexually transmitted infection
YPAG: young people advisory group
YPAGNE: young people advisory group north east

## References

[1] (2018). Adult Drinking Habits in Great Britain. Office for National Statistics.

[2] (2019). Smoking, Drinking and Drug Use among Young People in England. NHS Digital.

[3] (2015). Communications Market Report. OfCom.

[4] (2016). Children and Parents: Media Use and Attitudes Report. OfCom.

[5] McCambridge J, McAlaney J, Rowe R (2011). Adult consequences of late adolescent alcohol consumption: a systematic review of cohort studies. PLoS Med.

[6] Ellickson PL, Tucker JS, Klein DJ (2003). Ten-year prospective study of public health problems associated with early drinking. Pediatrics.

[7] Hall WD, Patton G, Stockings E, Weier M, Lynskey M, Morley KI, Degenhardt L (2016). Why young people's substance use matters for global health. Lancet Psychiatry.

[8] Behrendt S, Wittchen H, Höfler M, Lieb R, Beesdo K (2009). Transitions from first substance use to substance use disorders in adolescence: is early onset associated with a rapid escalation?. Drug Alcohol Depend.

[9] Hingson RW, Heeren T, Winter MR (2006). Age at drinking onset and alcohol dependence: age at onset, duration, and severity. Arch Pediatr Adolesc Med.

[10] Dawson DA, Goldstein RB, Chou SP, Ruan WJ, Grant BF (2008). Age at first drink and the first incidence of adult-onset DSM-IV alcohol use disorders. Alcohol Clin Exp Res.

[11] Fowler LA, Holt SL, Joshi D (2016). Mobile technology-based interventions for adult users of alcohol: a systematic review of the literature. Addict Behav.

[12] Rooke S, Thorsteinsson E, Karpin A, Copeland J, Allsop D (2010). Computer-delivered interventions for alcohol and tobacco use: a meta-analysis. Addiction.

[13] Dedert EA, McDuffie JR, Stein R, McNiel JM, Kosinski AS, Freiermuth CE, Hemminger A, Williams JW (2015). Electronic interventions for alcohol misuse and alcohol use disorders: a systematic review. Ann Intern Med.

[14] Kaner EF, Beyer FR, Garnett C, Crane D, Brown J, Muirhead C, Redmore J, O'Donnell A, Newham JJ, de Vocht F, Hickman M, Brown H, Maniatopoulos G, Michie S (2017). Personalised digital interventions for reducing hazardous and harmful alcohol consumption in community-dwelling populations. Cochrane Database Syst Rev.

[15] Champion KE, Newton NC, Teesson M (2016). Prevention of alcohol and other drug use and related harm in the digital age: what does the evidence tell us?. Curr Opin Psychiatry.

[16] Newton NC, Champion KE, Slade T, Chapman C, Stapinski L, Koning I, Tonks Z, Teesson M (2017). A systematic review of combined student- and parent-based programs to prevent alcohol and other drug use among adolescents. Drug Alcohol Rev.

[17] Bhochhibhoya A, Hayes L, Branscum P, Taylor L (2015). The use of the internet for prevention of binge drinking among the college population: a systematic review of evidence. Alcohol Alcohol.

[18] Black N, Mullan B, Sharpe L (2016). Computer-delivered interventions for reducing alcohol consumption: meta-analysis and meta-regression using behaviour change techniques and theory. Health Psychol Rev.

[19] Riper H, Hoogendoorn A, Cuijpers P, Karyotaki E, Boumparis N, Mira A, Andersson G, Berman AH, Bertholet N, Bischof G, Blankers M, Boon B, Boß L, Brendryen H, Cunningham J, Ebert D, Hansen A, Hester R, Khadjesari Z, Kramer J, Murray E, Postel M, Schulz D, Sinadinovic K, Suffoletto B, Sundström C, de Vries H, Wallace P, Wiers RW, Smit JH (2018). Effectiveness and treatment moderators of internet interventions for adult problem drinking: an individual patient data meta-analysis of 19 randomised controlled trials. PLoS Med.

[20] Wood SK, Eckley L, Hughes K, Hardcastle KA, Bellis MA, Schrooten J, Demetrovics Z, Voorham L (2014). Computer-based programmes for the prevention and management of illicit recreational drug use: a systematic review. Addict Behav.

[21] (2019). Using Digital Technology to Improve Learning. Education Endowment Foundation.

[22] (2019). Realising the Potential of Technology in Education: A Strategy for Education Providers and the Technology Industry. Department for Education UK.

[23] Field M, Campbell F, Hock E, Wong R (2019). Digital Interventions to Reduce Alcohol-Related Harm: A Rapid Horizon-Scanning Review. Alcohol Change UK.

[24] Davies EL (2018). Feasibility of the prototype willingness model as the basis for school-delivered alcohol misuse prevention: a qualitative think-aloud study to explore acceptability of 'the alcohol smart quiz' with adolescents and teachers. J Health Psychol.

[25] Davies EL, Martin J, Foxcroft DR (2015). Development and acceptability of a co-produced online intervention to prevent alcohol misuse in adolescents: a think aloud study. JMIR Hum Factors.

[26] Davies EL, Lonsdale AJ, Hennelly SE, Winstock AR, Foxcroft DR (2017). Personalized digital interventions showed no impact on risky drinking in young adults: a pilot randomized controlled trial. Alcohol Alcohol.

[27] Yardley L, Morrison L, Bradbury K, Muller I (2015). The person-based approach to intervention development: application to digital health-related behavior change interventions. J Med Internet Res.

[28] Michie S, Johnston M, Francis J, Hardeman W, Eccles M (2008). From theory to intervention: mapping theoretically derived behavioural determinants to behaviour change techniques. Appl Psychol.

[29] MacArthur GJ, Jacob N, Pound P, Hickman M, Campbell R (2017). Among friends: a qualitative exploration of the role of peers in young people's alcohol use using Bourdieu's concepts of habitus, field and capital. Sociol Health Illn.

[30] MacArthur GJ, Hickman M, Campbell R (2020). Qualitative exploration of the intersection between social influences and cultural norms in relation to the development of alcohol use behaviour during adolescence. BMJ Open.

[31] Crane D, Garnett C, Brown J, West R, Michie S (2017). Factors influencing usability of a smartphone app to reduce excessive alcohol consumption: think aloud and interview studies. Front Public Health.

[32] Braun V, Clarke V, Hayfield N (2019). Thematic analysis. Handbook of Research Methods in Health Social Sciences.

[33] Gale NK, Heath G, Cameron E, Rashid S, Redwood S (2013). Using the framework method for the analysis of qualitative data in multi-disciplinary health research. BMC Med Res Methodol.

[34] Glaser BG (1998). Doing Grounded Theory: Issues & Discussion.

[35] (2016). Smoking, Drinking and Drug Use Among Young People in England. NHS digital.

[36] Milward J, Deluca P, Drummond C, Watson R, Dunne J, Kimergård A (2017). Usability testing of the BRANCH smartphone app designed to reduce harmful drinking in young adults. JMIR Mhealth Uhealth.

[37] Rundle-Thiele S, Schuster L, Dietrich T, Russell-Bennett R, Drennan J, Leo C, Connor JP (2015). Maintaining or changing a drinking behavior? GOKA's short-term outcomes. J Bus Res.

[38] de Visser RO, Wheeler Z, Abraham C, Smith JA (2013). 'Drinking is our modern way of bonding': young people's beliefs about interventions to encourage moderate drinking. Psychol Health.

[39] Mercken L, Steglich C, Knibbe R, Vries HD (2012). Dynamics of friendship networks and alcohol use in early and mid-adolescence. J Stud Alcohol Drugs.

[40] Marton C, Choo CW (2012). A review of theoretical models of health information seeking on the web. J Doc.

[41] Horvath KJ, Ecklund AM, Hunt SL, Nelson TF, Toomey TL (2015). Developing Internet-based health interventions: a guide for public health researchers and practitioners. J Med Internet Res.

[42] Jander A, Crutzen R, Mercken L, de Vries H (2014). A Web-based computer-tailored game to reduce binge drinking among 16 to 18 year old Dutch adolescents: development and study protocol. BMC Public Health.

[43] Guest G, Bunce A, Johnson L (2006). How many interviews are enough?: an experiment with data saturation and variability. Field Methods.

