# Supplementary figures and images for: Qualitative Evaluation of Web-Based Digital Intervention to Prevent and Reduce Excessive Alcohol Use and Harm Among Young People Aged 14-15 Years: A “Think-Aloud” Study
Source: JMIR Pediatr Parent. 2020 Dec 15;3(2):e19749. doi: 10.2196/19749 (PMC7772065; doi:10.2196/19749)

Supplementary Material

Appendix 1: Screenshots of exemplar prototype web pages.


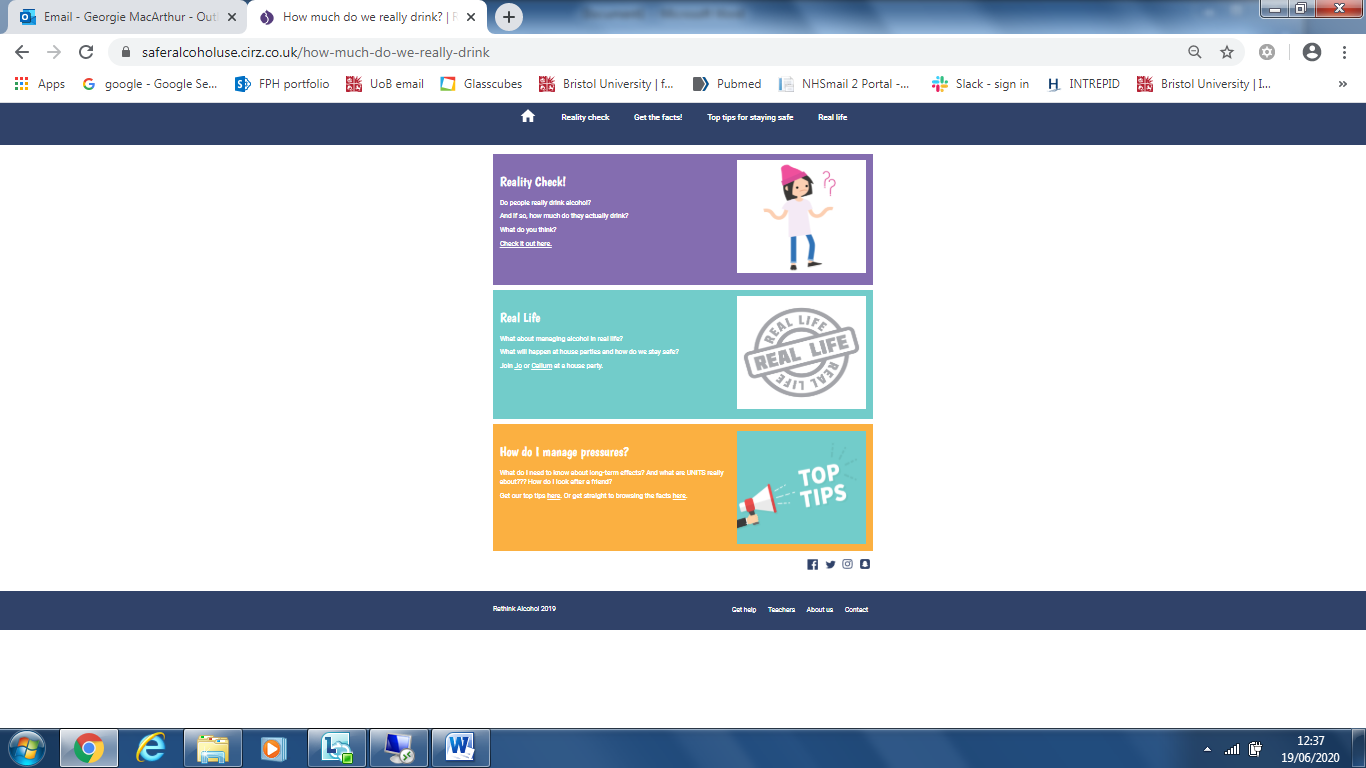

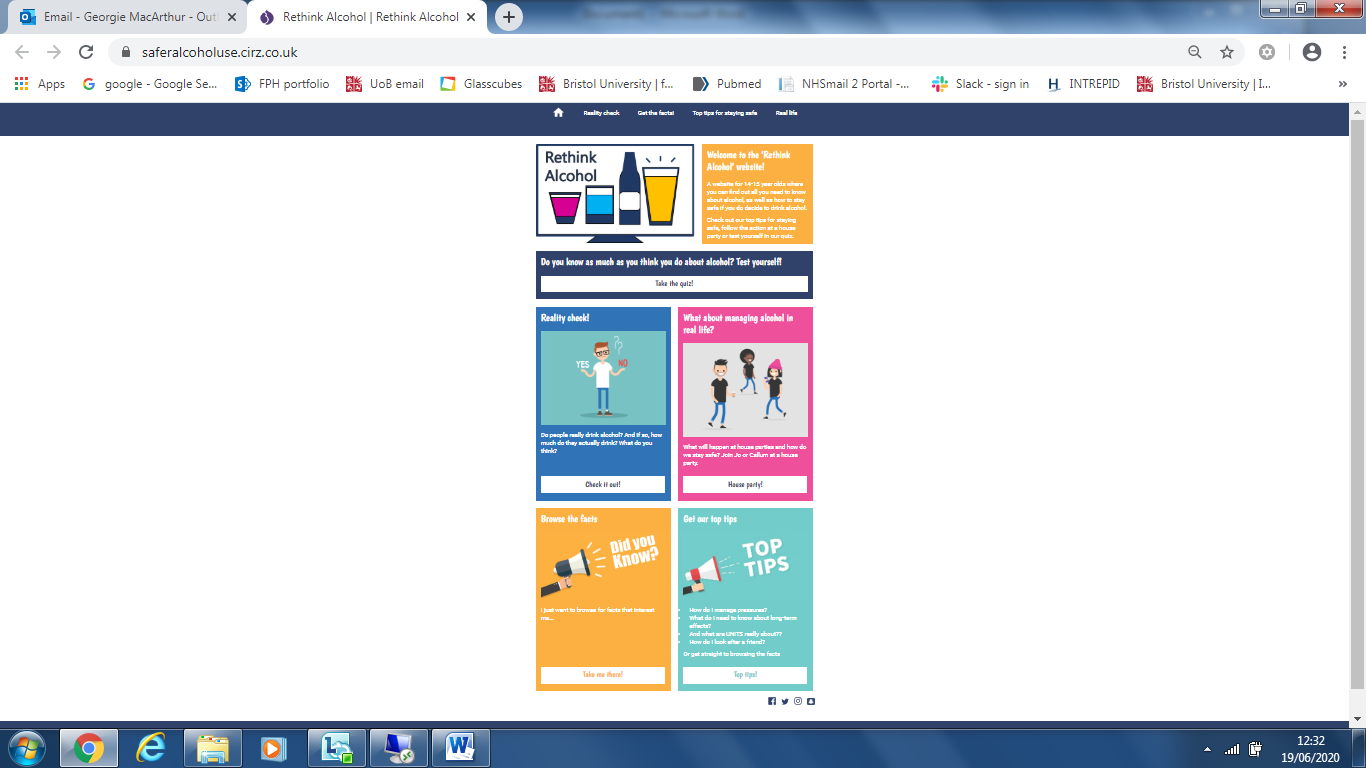

Supplement: Multimedia Appendix 1 [file pediatrics_v3i2e19749_app1.docx]
