# Supplementary material for: Qualitative Evaluation of Web-Based Digital Intervention to Prevent and Reduce Excessive Alcohol Use and Harm Among Young People Aged 14-15 Years: A “Think-Aloud” Study
Source: JMIR Pediatr Parent. 2020 Dec 15;3(2):e19749. doi: 10.2196/19749 (PMC7772065; doi:10.2196/19749)
Supplement: Multimedia Appendix 2 [file pediatrics_v3i2e19749_app2.docx]

Appendix 2: Themes and subthemes for Framework analysis.

| **Theme** | **Subtheme** |
| --- | --- |
| Content | Additional content ideas |
|  | Change or move content to a different area |
|  | General positive comments |
|  | General negative comments |
| Credibility of the website | Professional and consistent |
|  | Trustworthiness |
|  | Accurate |
| Design | Positive comments |
|  | General negative comments |
|  | Suggestions for change |
| Make the website easy to navigate | General positive comments |
|  | General negative comments |
|  | Home page |
|  | Number of clicks |
|  | How user-friendly the website is |
| Make the website easy to understand | Clarity |
|  | Getting information quickly |
|  | Level of complexity |
|  | Quantity |
| Tailor website to age group | General comments about the audience |
|  | Engagement, appeal and fun |
|  | Interactive elements |
|  | Wording, tone and perspective |
